# Influence of soft segment structure, hydrogen bonding, and diisocyanate symmetry on morphology and properties of segmented thermoplastic polyurethanes and polyureas

**DOI:** 10.55730/1300-0527.3591

**Published:** 2023-06-22

**Authors:** Emel YILGÖR, İskender YILGÖR

**Affiliations:** Chemistry Department, Koç University, İstanbul, Turkiye

**Keywords:** Polyurethane, polyurea, hydrogen bonding, morphology, diisocyanate

## Abstract

A comprehensive review of the structure-morphology-property relations in segmented thermoplastic polyurethanes and polyureas (TPU) is provided. Special emphasis is given to the influence of the soft segment structure, polarity, and molecular weight, diisocyanate symmetry and the nature, extent, and strength of hydrogen bonding on the morphology and thermal and mechanical properties of TPUs. Experimental results obtained on composition-dependent TPU morphology and properties by various techniques were also compared by the morphology profiles generated by computational methods such as quantum mechanical calculations and molecular dynamics simulations.

## 1. Introduction

**Figure 1:**
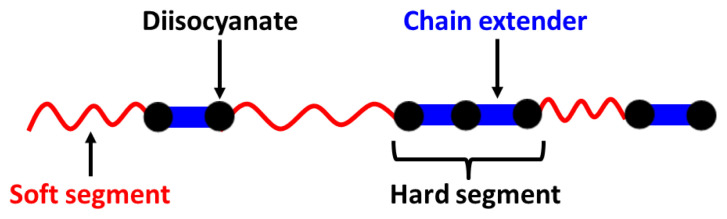
Schematic representation of the backbone structure of a linear TPU.

Segmented thermoplastic polyurethanes and polyureas (TPUs) are linear macromolecules consisting of alternating soft and hard segments covalently linked together by urethane or urea linkages, which is schematically shown in Figure 1. Soft segments (SS) are flexible oligomers with average molecular weights of 1000 to 5000 g/mol and very low glass transition temperatures (Tg) (−50 to −120 °C). Hard segments (HS) are generally the reaction products of a diisocyanate and a low molecular weight diol or diamine chain extender, which can make strong hydrogen bonding and/or crystalline. TPUs are one of the most important classes of synthetic polymers which find applications in different fields that include textile fibers, synthetic leather, biomaterials, functional coatings, adhesives, membranes, and many others. All of these make TPUs one of the most widely investigated polymeric materials by both academic and industrial researchers [1].

**Figure 2:**
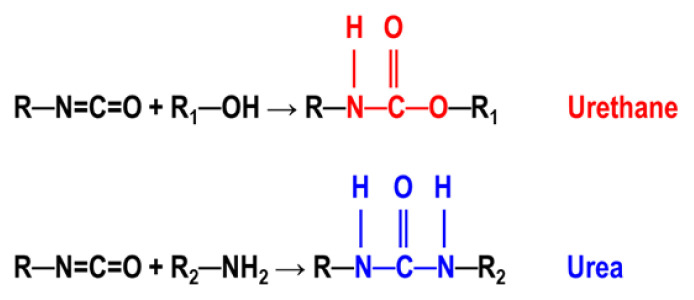
Reactions leading to the formation of urethane and urea linkages.

Urethane and urea groups are produced by the addition reaction of an alcohol (OH) or an amine (NH_2_) group to isocyanate (N=C=O) respectively, as shown in Figure 2. Since amines are much more nucleophilic than alcohols, urea formation reactions take place at room temperature, while for urethane formation reactions high temperatures and catalysts are needed [2].

**Figure 3:**
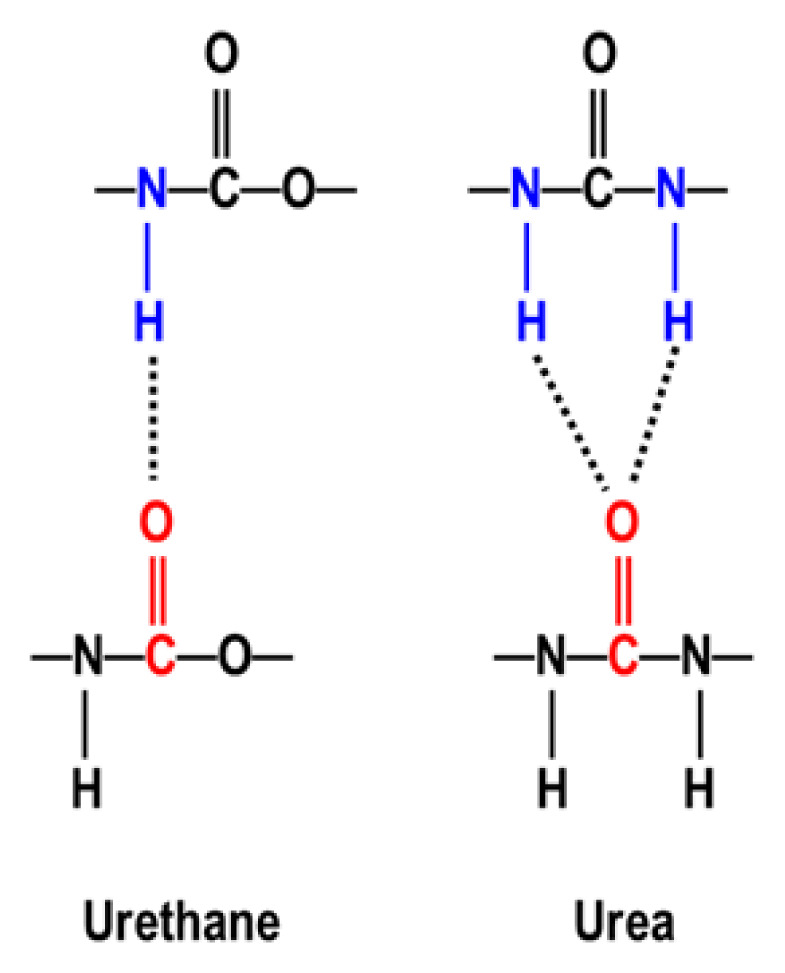
Schematic representation of monodentate and bidentate H-bonding between urethane and urea groups respectively.

One of the most important features of the urethane and urea groups is their intermolecular hydrogen bonding (H-bonding) capacities. As shown in Figure 3, while urethanes generally make monodentate H-bonding between N-H and C=O groups, urea groups can make much stronger bidentate H-bonding. H-bond energies determined by quantum mechanical calculations are 46.5 kJ/mol for urethane and 58.5 kJ/mol for urea groups [3]. H-bonding plays a major role in determining TPU morphology and properties, which is one of the main topics of this article.

The availability of a wide selection of commercially available starting materials allows the design and synthesis of an almost infinite number of TPUs with different structures, compositions, and properties. This creates a major challenge for the scientists investigating structure-property behavior of TPUs, since a very large number of parameters (listed below) must be considered and correlated to develop a comprehensive structure-morphology-property relationship [1]. Important parameters that influence the morphology and properties of TPUs are:

- chemical structure, average molecular weight, and solubility parameter or polarity of the soft segment,
- chemical structure and symmetry of the diisocyanate,
- type (diol or diamine) and chemical structure of the chain extender,
- structure (urethane or urea) and average chain length of the hard segments,
- strength and extent of hydrogen bonding (urethane versus urea) in hard segments, and
- soft/hard segment ratio in TPU.

As stated in the title of the manuscript, the main aim of this article is to understand and explain the influence of:

- soft segment structure, polarity, and average molecular weight,
- nature and extent of hydrogen bonding in the system, and
- diisocyanate structure and symmetry, on TPU morphology and properties.

**Figure 4:**
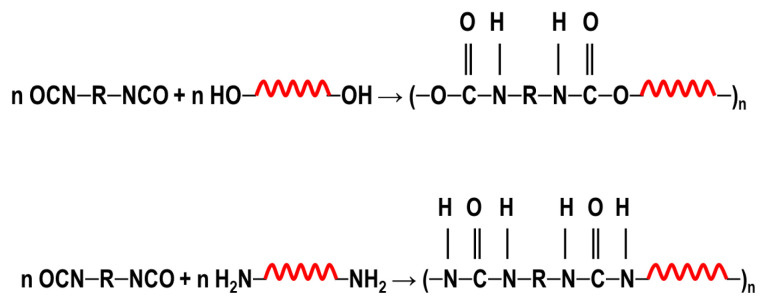
Preparation of segmented TPUs with uniform urethane and urea hard segments by the stoichiometric reactions of diisocyanates and hydroxy or amine-terminated soft segment oligomers. (R) indicates the diisocyanate backbone and the spring ( 
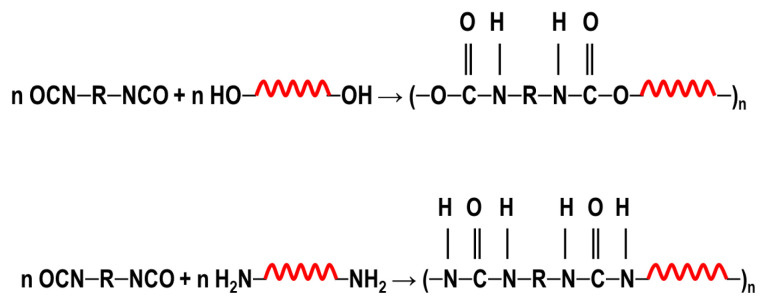
) indicates the soft segment backbone.

To achieve this, we also designed and synthesized model TPUs with well-defined structures by the stoichiometric reactions of various soft segment oligomers and diisocyanates [4–6] as shown in Figure 4. Chemical structures of the soft segment oligomers and diisocyanates used during the synthesis are provided in Table 1. TPUs with a large variety of soft and hard segments and chemical compositions have also been prepared by many other research groups to investigate structure-morphology-property relations in TPUs [7–11].

## 2. Influence of soft segment structure, polarity, and molecular weight on TPU morphology and properties

Soft segment structure and its polarity (Hildebrand or Hansen solubility parameter) play very critical roles in determining the extent and nature of microphase separation between hard and soft segments and TPU properties. The chemical structures of the most widely used soft segment oligomers are given in Table 1.

Hildebrand solubility parameter (δ), which can be estimated by using the equation given below, is a measure of the polarity of a molecule.


$$\delta ={\left((\Delta {\text{H}}_{\text{v}}-\text{RT})/{\text{V}}_{\text{M}}\right)}^{1/2}$$

where, ΔH_v_ is the enthalpy of vaporization, R gas constant, T absolute temperature, and V_M_ is the molar volume of the molecule.

If (δ) is low, the molecule is nonpolar if it is high, it is polar. Highly nonpolar n-pentane has the lowest solubility parameter of 14.4 (J/cm^3^)^1/2^, whereas highly polar water has a solubility parameter of 47.8 (J/cm^3^)^1/2^. Urethane (HNCOO) and urea (HNCONH) groups are highly polar with calculated solubility parameter (δ) values of 37.2 and 45.6 (J/cm^3^)^1/2^ respectively [12]. Just like water, they can make strong hydrogen bonding within themselves or with polar soft segments containing ester (COO), ether (COC), and carbonate (OCOO) group [3,12,13]. On the other hand, polydimethylsiloxane (PDMS) (Si(CH_3_)_2_O) and polyisobutylene (PIB) (CH_2_C(CH_3_)_2_) are highly nonpolar, with solubility parameter values of 15.6 and 16.4 (J/cm^3^)^1/2^ and cannot make hydrogen bonding with urethane or urea hard segments. Therefore, when PDMS or PIB are used as the soft segments, morphologies of the TPUs formed are expected to display excellent microphase separation, when compared with aliphatic polyether, polyester, and polycarbonate soft segments, which are expected to display some phase mixing.

**Figure 5:**
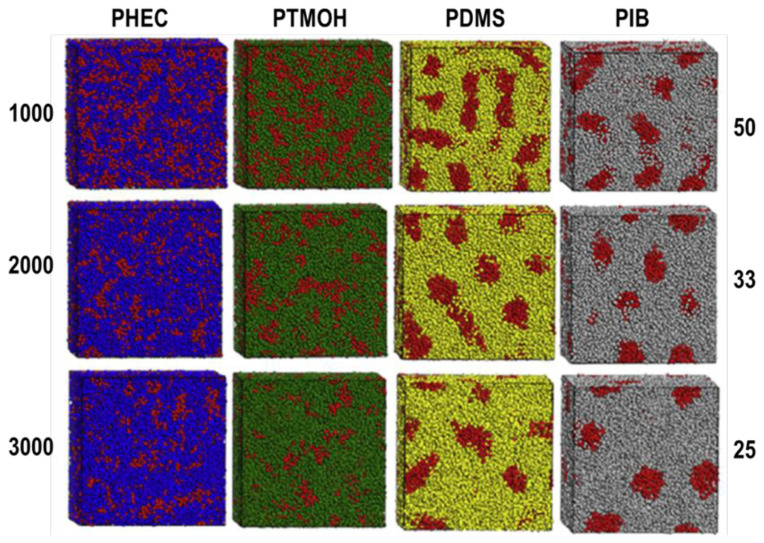
Simulated microphase morphologies of segmented TPUs with 1000 g/mol MDI+BD based hard segment and PHEC, PTMO, PDMS, and PIB soft segments with different molecular weights (shown on right-hand-side) and SS/HS contents (shown at left-hand-side). The red parts are Urethane HS [14] (cell dimensions 24 × 24 × 24 nm).

This has been clearly demonstrated by both experimental and computational studies on various polyurethanes and polyureas. In a computational study carried out by molecular dynamics simulations morphologies of TPUs based on polar poly(hexylethyl carbonate) (PHEC) and poly(tetramethylene oxide) (PTMO), and nonpolar polyisobutylene (PIB) and polydimethylsiloxane (PDMS) with molecular weights of 1000, 2000, and 3000 g/mol and hard segment contents of 25, 33, and 50 weight percent were generated and compared. In all TPUs, a model MDI-based urethane hard segment with an average molecular weight of 1000 g/mol was used [14]. Microphase morphologies of model TPUs generated are provided in Figure 5.

As can clearly be seen in Figure 5, and as expected, PDMS and PIB-based TPUs display excellent microphase separation, with well-defined urethane hard segment domains distributed homogeneously throughout the soft segment matrix. On the other hand, PHEC and PTMO-based TPUs display much poorer microphase separation, where urethane hard segments cannot form well-defined domains and are distributed randomly in the soft segment matrix. This is due to extensive intermolecular interaction between urethane hard segments with polar carbonate and ether groups present in soft segments, which leads to phase mixing. It is also interesting to note that with an increase in the molecular weight of the soft segment from 1000 to 3000 g/mol, especially PTMO based TPU shows noticeable improvement in microphase separation. This is most probably due to a lower entropy of mixing because of the formation of fewer covalent bonds between HS and SS as the molecular weight of PTMO increases.

Experimental investigation of the morphologies of PHEC, PTMO, and PDMS based TPUs were conducted by Runt and coworkers [10]. They synthesized homologous TPUs using PHEC, PTMO, and PDMS soft segment oligomers with an average molecular weight of 1000 g/mol which had 40% by weight urethane hard segments based on MDI and 1,4-butanediol (BD). Using thermal, spectroscopic, and small angle x-ray scattering (SAXS) measurements they estimated the degree of microphase separation to be 0.13 for PHEC, 0.29 for PTMOH, and 1.0 for PDMS-based TPU. These results obtained from experimental techniques are very similar to those generated by molecular dynamics simulations discussed above [14].

Influence of the structure and polarity of the aliphatic polyether soft segments [(CH_2_)_x_-O)_n_] on the microphase morphologies of TPUs containing 55% by weight MDI+BD hard segments with an average molecular weight of 1000 g/mol were investigated [15]. For this purpose, polyether soft segments with molecular weights of 1000 g/mol were used but the number of methylene groups in the oligomers (x) was varied as follows: x = 2, 4, 6, 10, 12 [15]. As the number of methylene groups (x) increased, the polarity of the polyether decreased. This led to significant improvement in the extent of microphase separation in the homologous TPUs.

**Figure 6:**
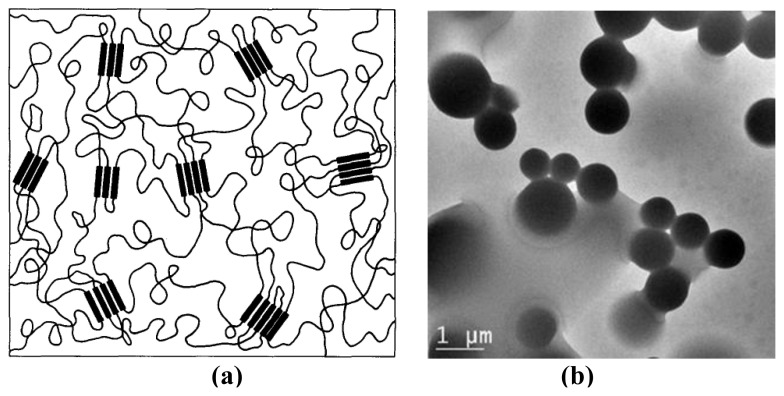
(a) Predicted microphase morphology for PDMS-urea copolymers, where uniform urea HS domains are randomly distributed in PDMS matrix with a very sharp interface between the two phases [6]. (b) TEM image of PDMS2k-urea thin film stained with CoCl_2_, showing the polymer morphology consisting of spherical urea domains distributed in a PDMS matrix, with a very sharp interface between two phases.

We synthesized thermoplastic PDMS-urea copolymers by the stoichiometric reactions of aminopropyl-terminated PDMS oligomers and MDI or HMDI [5,15,16]. We also investigated thermal, mechanical, and morphological behavior of these materials by various techniques. From these studies, we predicted a microphase morphology for these systems, consisting of well-defined and uniform urea HS domains randomly distributed in PDMS matrix with a very sharp interface between the two phases [6], as shown in Figure 6a. Many years later, we confirmed the presence of such a morphology, with a very sharp interface between PDMS and urea phases by transmission electron microscopy (TEM) studies as shown in the image given in Figure 6b. To obtain the TEM image we dissolved PDMS2k-urea copolymer in tetrahydrofuran (THF) and added CoCl_2_ as a staining agent. Highly polar CoCl_2_ only interacted with the urea hard segment domains and increased the electronic contrast significantly in the thin film produced, enabling us to obtain these morphological images.

## 3. Effect of hydrogen bonding on TPU morphology and properties

The nature and extent of hydrogen bonding between urethane or urea hard segments and competitive hydrogen bonding between hard and soft (polyether, polyester, polycarbonate, etc.) segments also play key roles in determining the morphology and properties of TPUs. To have a better understanding of the strengths of hydrogen bonding between hard-hard and hard-soft segments, H-bond energies of various segmental interactions calculated by quantum mechanical calculations are tabulated in Table 2 [3,13]. As expected, urea-urea has the highest H-bond energy, followed by urethane-urethane. Very interestingly, H-bond interactions between urethane and urea hard segments and ester and ether hard segments are also reasonably strong and H-bond energies are fairly high. On the other hand, no significant interaction is observed between siloxane and urea hard segments. These results clearly indicate that choice of the soft segment type and content has a significant effect on the extent of HS-HS and HS-SS H-bond interactions. This in turn will strongly influence the TPU morphology and properties as discussed below.

In the previous section where we discussed the influence of the number of methylene units in polyether structure on TPU morphology is also a good example of competitive hydrogen bonding between HS-HS and HS-SS. As the number of methylene groups in the SS oligomer increases, the number of ether groups decreases. This results in a decrease in the competitive urethane-ether H-bond interaction and improved microphase separation in the system.

We also investigated the influence of hydrogen bonding on the morphology and properties of PDMS-based TPUs. PDMS containing copolymers with similar chemical compositions were prepared by the stoichiometric reactions of α,ω-aminopropyl, α,ω-N-methylaminopropyl and α,ω-hydroxyhexyl terminated PDMS oligomers with HMDI, which resulted in urea, N-methylurea and urethane hard segments with different H-bond capacities [12]. Chemical compositions and stress-strain properties of silicone copolymers produced are provided in Table 3.

It is interesting to note in Table 3 that PDMS-urethane copolymers (PSUT) which have the lowest H-bond capacity, could not make solid films. They could only produce soft sticky films, which did not display any mechanical integrity. On the other hand, silicone-urea (PSU) and silicone-N-methylurea copolymers (PSMU) both made solid films. PSU copolymers displayed much higher modulus and tensile strength values when compared with their N-methyl substituted homologs. N-methyl substitution of urea groups prevented the formation of strong bidentate H-bonding, thus significantly reducing the H-bonding capacity of the urea groups and the tensile properties of homologous TPUs.

A detailed study on the synthesis and characterization of silicone-urethane and silicone-urea copolymers with a wide range of compositions was also performed [17]. The influence of PDMS molecular weight, HS content, and H-bond strength on morphologies and various thermal and mechanical properties of the resultant copolymers were investigated. It was demonstrated that when compared with silicone-urethane copolymers of similar structures and compositions silicone-urea copolymers with much stronger H-bonding capacity displayed better microphase separation, higher modulus and tensile strength, and much superior thermomechanical properties over a wider temperature range.

Furthermore, the influence of H-bond strength on PTMO-urethane and PTMO-urea copolymers with similar compositions was also investigated. As shown in Figure 4, model TPUs with uniform hard segments were synthesized by the stoichiometric reactions of PTMO and PTMN oligomers and four different asymmetric diisocyanates (MDI, HMDI, TDI, and MPDI) to produce homologous PTMO-urethane and PTMO-urea copolymers. While all polyureas formed solid films with good mechanical strength, none of the polyurethanes were able to form solid films. This we believe was mainly due to strong hydrogen bonding between urea hard segments but limited competitive H-bonding between urea and ether groups, which resulted in good microphase separation and film properties in PTMO-urea copolymers. On the other hand, weaker H-bonding between urethane groups which resulted in extensive competitive urethane-ether interaction prevented microphase separation in PTMO-urethanes investigated. Small angle x-ray scattering (SAXS), dynamic mechanical analysis (DMA), and atomic force microscopy (AFM) studies showed good microphase separation in PTMO-urea copolymers but did not indicate any microphase separation in PTMO-urethanes [18].

Fourier Transform Infrared Spectroscopy (FTIR) is a simple but effective technique for the quantitative determination of hydrogen bonding interactions in TPUs. This is due to well-defined peak positions of free N-H and C=O absorptions and extensive peak shifts observed due to hydrogen bonding [19,20]. Characteristic infrared absorption frequencies for urethane and urea groups under different H-bonding conditions are provided in Table 4.

**Figure 7:**
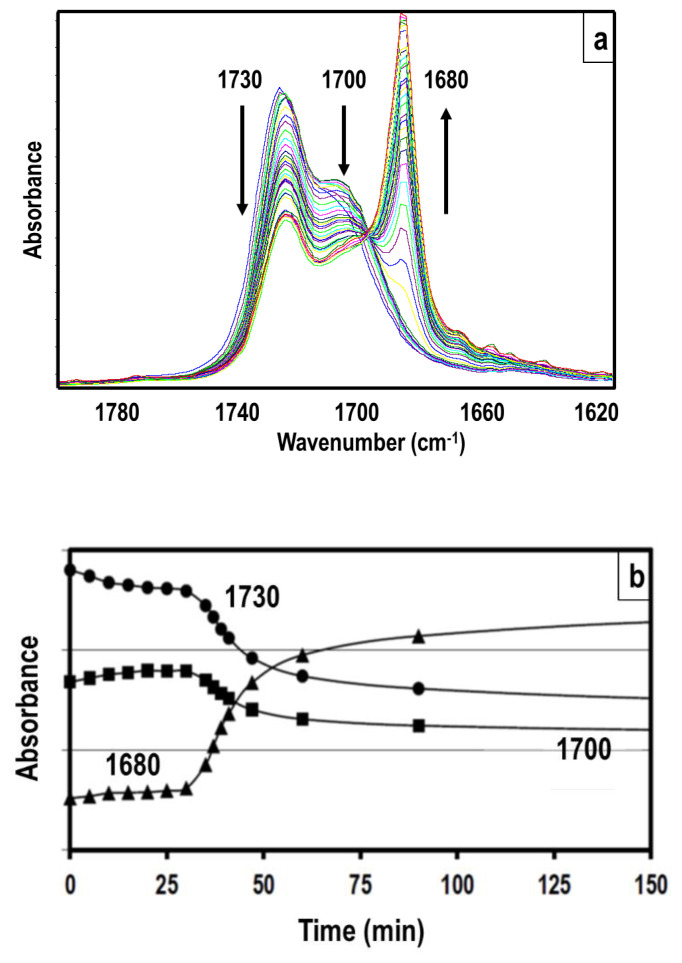
Time-dependent changes in various absorption peaks in the carbonyl region of the FTIR spectrum of HDI-PTMO1k polyurethane thin film, which was melted at 150 °C and then cooled at room temperature to reach equilibrium morphology [22].

As can clearly be seen in this Table, strong peak shifts are observed in N–H and C=O absorption bands due to hydrogen bonding. This information can be used to determine the extent of hydrogen bonding and get an estimate of the microphase separation in TPUs [21]. It is also possible to follow time-dependent microphase separation in TPU films as they are cooled down from melt. Figure 7a gives time-dependent changes in various absorption peaks in the carbonyl region of the FTIR spectrum of HDI-PTMO1k polyurethane thin film, which was cast on KBr disc and kept at 150 °C for 30 min to get a completely molten and phase mixed system [22]. After removing from the 150 °C oven the sample was kept at room temperature and FTIR spectra were obtained as a function of time until the equilibrium morphology was attained, or until no change was observed in peak absorptions.

As can be seen in Figure 7a significant changes in the absorbance values of three different C=O peaks were observed. These were nonhydrogen bonded C=O peak at 1730 cm^−1^, weakly hydrogen-bonded shoulder at 1700 cm^−1^ and strongly hydrogen bonded C=O peak at 1680 cm^−1^. As can clearly be seen in Figure 7a, strongly hydrogen bonded carbonyl peak did not exist in the molten film since it was totally phase mixed. As the film cooled down, hydrogen bonding between urethane groups took place and the absorbance value of 1680 cm^−1^ peak increased significantly. On the other hand, there was a dramatic drop in the absorbance value of 1730 cm^−1^ peak over time as free or nonhydrogen bonded C=O groups were converted to strongly hydrogen bonded units. A slow decrease in the magnitude of 1700 cm^−1^ peak is also observed over time as these weakly H-bonded C=O groups also strongly H-bonded with time. Time-dependent changes in the absorbance values of all peaks are plotted in Figure 7b, where absorbance values seem to level off and reach a plateau value in about 150 min, indicating the establishment of the equilibrium morphology for the HDI-PTMO1k polyurethane system.

**Figure 8:**
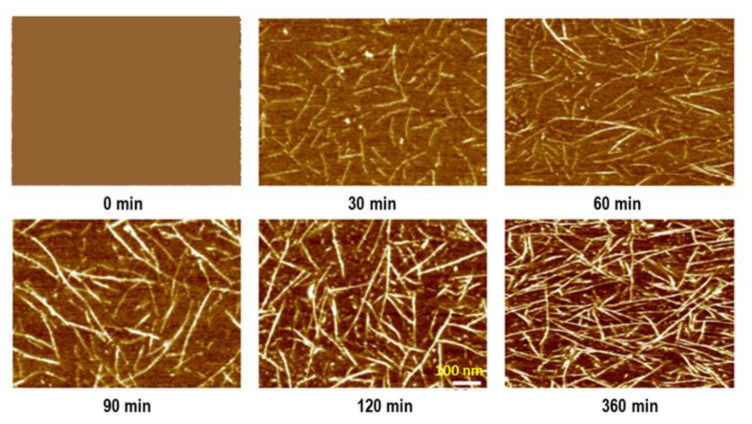
AFM images demonstrating time-dependent microphase separation and morphology development in molten PPDI-PTMO1k polyurethane films at room temperature [20].

The morphology development of PPDI-PTMO1k polyurethane was also monitored by AFM, where a thin film of the PPDI-PTMO1k copolymer was melted by heating to 70 °C (just above its melting point) and then kept at room temperature [20,23]. As the sample cooled down AFM images given in Figure 8 were recorded over time, where yellow-colored ribbons indicate the urethane hard segments.

As can be seen in these AFM images, molten PPDI-PTMO1k polyurethane film has no features, indicating a complete phase mixed morphology. After 30 min, as a result of strong hydrogen bonding between urethane groups fibrillar hard segments are observed, clearly indicating the start of microphase separation. As expected, with time the fibrillar urethane hard segment content of the films increased and after 360 min a well-defined microphase separated hard segment network was formed.

## 4. Role of diisocyanate chain symmetry on TPU morphology and properties

One of the most interesting parameters that influence TPU morphology and properties is the symmetry of the diisocyanate. Unfortunately, this aspect of polyurethane chemistry has not been investigated in detail. This is most probably due to the large-scale commercial use of MDI, TDI, HMDI, and IPDI in TPU production, which strongly influenced both academic and industrial researchers to design, synthesize, and characterize TPUs using these diisocyanates and investigate their structure-morphology-property relations. We demonstrated that when symmetrical diisocyanates, such as PPDI, HDI, and CHDI are used, TPUs produced displayed much stronger H-bonding, better microphase separation, and significantly higher mechanical strength and superior thermal properties, when compared with those based on MDI, TDI, and HMDI [1,4,18,22].

**Figure 9:**
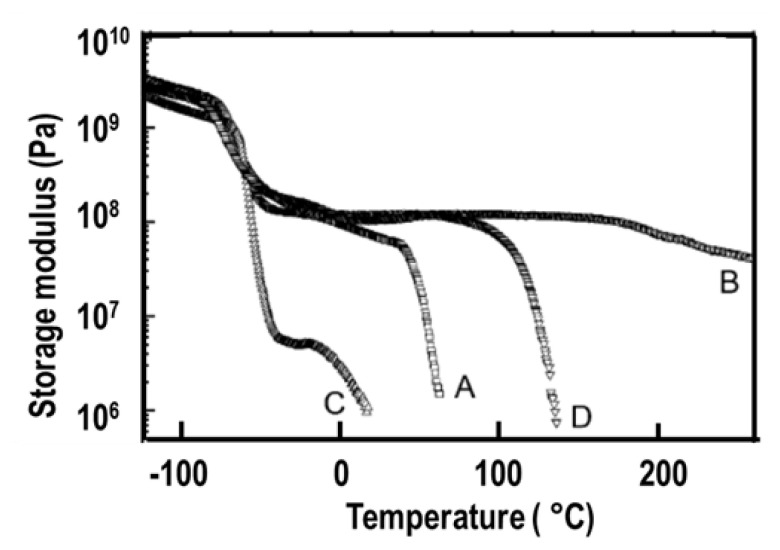
Modulus-temperature curves for TPUs prepared through stoichiometric reactions of PPDI and MPDI with PTMO1k and PTMN1k. (A) PPDI-urethane, (B) PPDI-urea, (C) MPDI-urethane, and (D) MPDI-urea [4].

To further demonstrate the dramatic influence of diisocyanate chain symmetry (and hydrogen bonding) on the morphology and properties, polyurethane and polyurea copolymers with similar compositions were prepared by the stoichiometric reactions of symmetrical PPDI and unsymmetrical MPDI with PTMO1k and PTMN1k soft segments. Comparative modulus-temperature curves for these TPUs are provided in Figure 9 [4]. Since all polymers are based on the same soft segment and have similar compositions, they all show a well-defined SS glass transition around −70 °C. Interestingly, after the SS glass transition, depending on the diisocyanate symmetry and type of the hard segment (urethane or urea) rubbery plateaus show significantly different behavior. Asymmetric MPDI-urethane, which only forms a very weak sticky film, shows almost no rubbery plateau and flows below room temperature. On the other hand, symmetric PPDI-urethane, which makes a good solid film, shows a well-defined rubbery plateau extending up to about 60 °C. Both MPDI-urea and PPDI-urea copolymers, due to very strong urea H-bonding in their hard segments, display extended rubbery plateaus with PPDI-urea going well above 200 °C. These results also indicate the presence of microphase-separated morphologies in PPDI-urethane and urea and MPDI-urea copolymers.

Diisocyanate symmetry and hydrogen bond strength also contribute significantly to the stress-strain behavior of TPUs. Chemical compositions and tensile properties of PPDI, MPDI, PTMO1k, and PTMN1k polyureas and polyurethanes are provided in Table 5 [18]. As can be seen in this Table, while asymmetric MPDI-based polyurethane does not possess any mechanical strength, symmetric PPDI-based polyurethane shows good elastomeric properties. Similarly, symmetric PPDI-urea shows much superior tensile properties when compared with its asymmetric MPDI based homolog. All these results clearly demonstrate the significant role played by diisocyanate symmetry on TPU morphology and properties.

## 5. Conclusions

Thermoplastic polyurethanes and polyureas (TPUs) are versatile materials which can display properties ranging from soft rubbers to tough plastics, depending on their chemical compositions and microphase morphologies. Commercial availability of an extremely wide selection of starting materials allows scientists to tailor design and synthesize a very large number of TPUs. Many parameters which include soft segment structure, polarity, molecular weight, diisocyanate structure and symmetry and the nature and strength of the hydrogen bonding within the hard segments strongly influence morphological behavior and physical and chemical properties of TPUs. In this article, a comprehensive review of the influence of these parameters on the structure-morphology-property behavior of segmented thermoplastic polyurethanes and polyureas is provided.

## Figures and Tables

**Table 1:** Chemical structures of the soft segment oligomers and diisocyanates.

Name	Code	Chemical structure
Aminopropyl polydimethylsiloxane	PDMS	H_2_N–(CH_2_)_3_–(Si(CH_3_)_2_O)_n_Si(CH_3_)_2_–(CH_2_)_3_–NH_2_
Polyisobutylene glycol	PIB	HO–(CH_2_–C(CH_3_)_2_)_n_–OH
Aminopropyl Poly(tetramethylene oxide)	PTMN	H_2_N–(CH_2_)_3_–O–(CH_2_–CH_2_–CH_2_–CH_2_–O)_n_–(CH_2_)_3_–NH_2_
Poly(tetramethylene oxide glycol	PTMO	HO–(CH_2_–CH_2_–CH_2_–CH_2_–O)_n_–H
Poly(hexylethyl carbonate) glycol	PHEC	HO-((CH_2_)_2_–OCOO(CH_2_)_6_)_n_–OH
1,4-Phenylene diisocyanate	PPDI	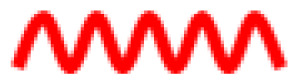
1,3-Phenylene diisocyanate	MPDI	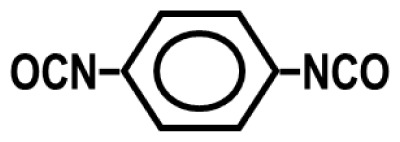
1,6-Diisocyanatohexane	HDI	OCN-(CH_2_)_6_-NCO
Trans-1,4-cyclohexyl diisocyanate	CHDI	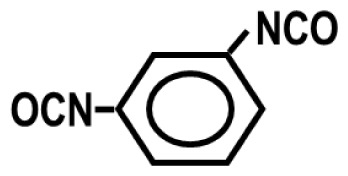
Bis(4-isocyanato cyclohexyl) methane	HMDI	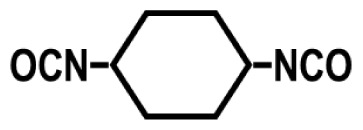
Bis(p-isocyanatophenyl) methane	MDI	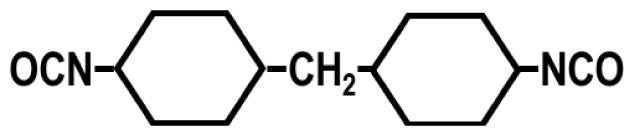
Isophorone diisocyanate	IPDI	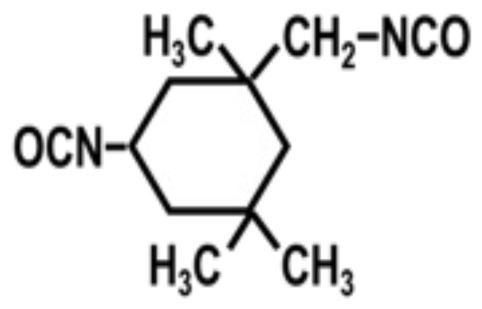

**Table 2:** Hydrogen bond energies and bond lengths, determined by QMC [3,13].

Interaction type	H-Bond energy (kJ/mol)	H-bond length (Å)
Urea-urea	58.5	1.83
Urethane-urethane	46.5	1.87
Urea-ester	29.7	2.20
Urea-ether	29.4	2.19
Urethane-ester	25.9	2.00
Urethane-ether	23.6	1.96
Urea-siloxane	7.5	2.78

**Table 3:** Compositions and tensile properties of PDMS copolymers with urea (U), N-methylurea (MU), and urethane (UT) hard segments [12].

Polymer code	PDMS M_n_ (g/mol)	HS type	HS content (wt%)	Modulus (MPa)	Tensile Strength (MPa)	Elongation (%)
PSU-1	900	U	32.8	157	20.1	430
PSU-2	2500	U	13.8	6.9	7.9	900
PSMU-1	900	MU	34.9	36	8.1	650
PSMU-2	2400	MU	15.3	4.8	2.1	750
PSUT-1	900	UT	39.4	--	--	--
PSUT-2	2400	UT	17.4	--	--	--

**Table 4:** Characteristic IR absorption frequencies of urethane and urea groups as a function of hydrogen bonding.

Group	Mode	Wavenumber (cm^−1^)
N–H	Free	3445–3450
N–H	N–H⋯N–H	3315–3340
N–H	N–H⋯O (ether)	3260–3290
C=O (urethane)	Free	1730–1740
C=O (urethane)	C=O⋯H–N	1680–1710
C=O (urea)	Free	1690–1700
C=O (urea)	C=O⋯H–N (disordered)	1660–1670
C=O (urea)	C=O⋯H–N (ordered)	1630–1645

**Table 5:** Influence of diisocyanate symmetry on tensile properties of homologous polyurethane and polyurea copolymers [18].

Polymer code	HS content (wt%)	Young’s modulus (MPa)	Tensile strength (MPa)	Elongation at break (%)
PPDI-Urethane	14.1	30.0	14.1	950
MPDI-urethane	14.1	--	--	--
PPDI-Urea	12.7	75.6	19.5	540
MPDI-urea	12.7	63.1	5.40	700

## References

[1] Yilgör I, Yilgör E, Wilkes GL (2015). Critical parameters in designing segmented polyurethanes and their effect on morphology and properties: A comprehensive review. Polymer.

[2] Hepburn C (1992). Polyurethane elastomers.

[3] Yilgör E, Yilgör I, Yurtsever E (2002). Hydrogen bonding and polyurethane morphology I. Quantum mechanical calculations of hydrogen bond energies and vibrational spectroscopy of model compounds. Polymer.

[4] Sheth JP, Klinedinst DB, Wilkes GL, Yilgör I, Yilgör E (2005). Role of chain symmetry and hydrogen bonding in segmented copolymers with monodisperse hard segments. Polymer.

[5] Yilgör I, Riffle JS, Wilkes GL, McGrath JE (1982). Siloxane-urea segmented copolymers: 1. Synthesis and characterization of model polymers from MDI and α, ω-bis(aminopropyl)polydimethylsiloxane. Polymer Bulletin.

[6] Tyagi D, Yilgör I, McGrath JE, Wilkes GL (1984). Segmented organosiloxane copolymers: 2. Thermal and mechanical properties of siloxane-urea copolymers. Polymer.

[7] Prisacariu C (2011). Polyurethane elastomers.

[8] Krol P (2007). Synthesis methods, chemical structures and phase structures of linear polyurethanes. Properties and applications of linear polyurethanes in polyurethane elastomers, copolymers and ionomers. Progress in Materials Science.

[9] Lamba NMK, Woodhouse KA, Cooper SL (2017). Polyurethanes in biomedical applications.

[10] Fragiadakis D, Runt J (2013). Molecular dynamics of segmented polyurethane copolymers: Influence of soft segment composition. Macromolecules.

[11] Datta J, Kasprzyk P (2018). Thermoplastic polyurethanes derived from petrochemical or renewable resources: A comprehensive review. Polymer Engineering and Science.

[12] Yilgör E, Yilgör I (2001). Hydrogen bonding: A critical parameter in designing silicone copolymers. Polymer.

[13] Yilgör E, Burgaz E, Yurtsever E, Yilgör I (2000). Comparison of hydrogen bonding in polydimethylsiloxane and polyether-based urethane and urea copolymers. Polymer.

[14] Yildirim E, Yurtsever M, Wilkes GL, Yilgör I (2016). Effect of Intersegmental interactions on the morphology of segmented polyurethanes with mixed soft segments: A coarse grained simulation study. Polymer.

[15] Martin DJ, Meijs GF, Renwick GM, Gunatillake PA, McCarthy SJ (1996). Effect of soft segment CH/O ratio on morphology and properties of a series of polyurethane elastomers. Journal of Applied Polymer Science.

[16] Tyagi D, Wilkes GL, Yilgör I, McGrath JE (1982). Siloxane-urea segmented copolymers: 2. Investigation of mechanical behavior. Polymer Bulletin.

[17] Sheth JP, Aneja A, Wilkes GL, Yilgör E, Atilla GE (2004). Influence of system variables on the morphological and dynamic mechanical behavior of polydimethylsiloxane based segmented polyurethane and polyurea copolymers: A comparative perspective. Polymer.

[18] Das S, Cox DF, Wilkes GL, Klinedinst DB, Yilgör I (2007). Effect of symmetry and H-Bond strength of hard segments on the structure-property relationships of segmented, nonchain extended polyurethanes and polyureas. Journal of Macromolecular Science, Part B: Physics.

[19] Yilgör I, Yilgör E, Das S, Wilkes GL (2009). Time-dependent morphology development in segmented polyetherurea copolymers based on aromatic diisocyanates. Journal of Polymer Science B Polymer Physics.

[20] Sheth JP, Klinedinst DB, Pechar TW, Wilkes GL, Yilgör E (2005). Time-dependent morphology development in a segmented polyurethane with monodisperse hard segments based on 1,4-phenylene diisocyanate. Macromolecules.

[21] Yilgör I, Yilgör E, Guler IG, Ward TC, Wilkes GL (2006). FTIR investigation of the influence of diisocyanate symmetry on the morphology development in model segmented polyurethanes. Polymer.

[22] Yilgör I, Yilgör E (2007). Structure-morphology-property behavior of segmented thermoplastic polyurethanes and polyureas prepared without chain extenders. Polymer Reviews.

[23] Klinedinst DB, Yilgör E, Yilgör I, Beyer FL, Sheth JP (2005). Structure-property behavior of new segmented polyurethanes and polyureas without use of chain extenders. Rubber Chemistry and Technology.

